# Personal

**Published:** 1909-12

**Authors:** 


### PERSONAL.

Brevet Lieutenant Colonel A. H. Briggs, M. D., Surgeon 65th Regiment N. G. N. Y., of Buffalo, was tendered a complimentary dinner by the officers of the regiment at the Saturn Club, Wednesday evening, October 27, 1909. Several appropriate speeches were made, under the guidance of General S. M. Welch, as toastmaster, and Dr. Briggs gave an interesting talk on his experiences during thirty years of continuous service with the sixty-fifth.

Dr. W. Scott Renner, has removed from 361 Pearl Street to 341 Linwood Avenue. Hours: 9 to 1 except Sundays. Special hours by appointment. Telephone, Bryant 1565.

Dr. A. B. Knisley, of Buffalo, announces that he has changed his office from No. 56 Woodlawn Avenue to No. 30 Woodlawn Avenue. Hours, 8-9, 1-3 and 7-8. Telephones: Bell, Bryant 574; Frontier 1602.

Dr. James A. Gardner, of Buffalo, read a paper on November 24, before the New York Society of the American Urological Association, entitled "A Case of Acute Fulminating Gangrene of the Penis and Scrotum with Known Port of Entry."

Dr. Charles E. Long, of Buffalo, has been appointed an examiner in midwifery for Erie County, in place of Dr. Ludwig Schroeter, resigned. The examiners in midwifery are appointed by the County Judge.

Dr. Morris N. Bemus, of Jamestown, has been appointed a member of the Board of Health of that city, in place of Dr. Jason Parker, resigned.

Dr. L. Bradley Dorr, of Buffalo, was chosen as a member of the city council at the last election. The medical profession will have a good friend in the council, in the person of Dr. Dorr.

